# The omicron (B.1.1.529) SARS-CoV-2 variant of concern does not readily infect Syrian hamsters

**DOI:** 10.1016/j.antiviral.2022.105253

**Published:** 2022-02

**Authors:** Rana Abdelnabi, Caroline S. Foo, Xin Zhang, Viktor Lemmens, Piet Maes, Bram Slechten, Joren Raymenants, Emmanuel André, Birgit Weynand, Kai Dallmeier, Johan Neyts

**Affiliations:** a KU Leuven Department of Microbiology, Immunology and Transplantation, Rega Institute for Medical Research, Laboratory of Virology and Chemotherapy, B-3000, Leuven, Belgium; b Molecular Vaccinology and Vaccine Discovery, Leuven, Belgium; c Laboratory of Clinical and Epidemiological Virology, Rega Institute, KU Leuven, Department of Microbiology, Immunology and Transplantation, 3000, Leuven, Belgium; d Zoonotic Infectious Diseases Unit, Leuven, Belgium; e Department of Laboratory Medicine, UZ Leuven Hospital, 3000, Leuven, Belgium; f Laboratory of Clinical Bacteriology and Mycology, Department of Microbiology, Immunology and Transplantation, Rega Institute, KU Leuven, 3000, Leuven, Belgium; g KU Leuven Department of Imaging and Pathology, Translational Cell and Tissue Research, Division of Translational Cell and Tissue Research, B-3000, Leuven, Belgium; h Global Virus Network, GVN, Leuven, Belgium

**Keywords:** COVID-19, SARS-CoV-2 VoC, Omicron, Hamsters, Infectivity

## Abstract

The emergence of SARS-CoV-2 variants of concern (VoCs) has exacerbated the COVID-19 pandemic. End of November 2021, a new SARS-CoV-2 variant namely the omicron (B.1.1.529) emerged. Since this omicron variant is heavily mutated in the spike protein, WHO classified this variant as the 5th variant of concern (VoC). We previously demonstrated that the ancestral strain and the other SARS-CoV-2 VoCs replicate efficiently in and cause a COVID19-like pathology in Syrian hamsters. We here wanted to explore the infectivity of the omicron variant in comparison to the ancestral D614G strain in the hamster model. Strikingly, in hamsters that had been infected with the omicron variant, a 3 log_10_ lower viral RNA load was detected in the lungs as compared to animals infected with D614G and no infectious virus was detectable in this organ. Moreover, histopathological examination of the lungs from omicron-infected hamsters revealed no signs of peri-bronchial inflammation or bronchopneumonia.

## Highlights

• End of Nov 2021, WHO classified the omicron variant as a variant of concern.
• The omicron SARS-CoV-2 variant does not replicate in Syrian hamsters lungs.
• There are no signs of COVID19-like pathology in lungs of omicron-infected hamsters.

## 1 Main text

Variants of SARS-CoV-2 are still emerging in different parts of the world, posing a new public health threat. Even in highly endemic regions, some of these variants have replaced the formerly dominant strains and resulted in new waves of infections and new spikes in mortality (Plante et al., 2021). On November 24, 2021, South Africa officially reported the emergence of B.1.1.529 (omicron) variant to WHO. Two days later, the omicron variant has been classified by WHO as the 5th variant of concern (VoC) following the alpha, beta, gamma and delta VoCs (“World Health Organization., 2021 Classification of Omicron (B.1.1.529): SARS-CoV-2 Variant of Concern. November 26, 2021 (2021),” n.d.). Among these VoC, the omicron variant carries the highest number of spike protein mutations (>30 mutations) (Karim and Karim, 2021). Some of the spike mutations carried by the omicron variant have been reported in other VoCs to be associated with immune escape and reduced susceptibility to monoclonal antibodies (Karim and Karim, 2021). In addition, the omicron variant carries some spike mutations that could be involved in increased transmissibility, which is also supported by the rapid replacement of delta variant by omicron as the dominant variant in South Africa (del Rio et al., 2021; Karim and Karim, 2021). Fortunately there is now growing evidence that omicron causes a less severe pathology in man than the ancestral strains and the other VoC. We and others previously demonstrated that alpha, beta, gamma and delta VoCs are replicating efficiently in the lungs of Syrian hamsters and to a similar level as the ancestral strains (i.e. Wuhan and D614G strains) (Abdelnabi et al., 2021a, 2021b; Sharma et al., 2021). We here compare the infectivity of the omicron variant *versus* the ancestral D614G strain in our Syrian hamster model.

**Fig. 1:**
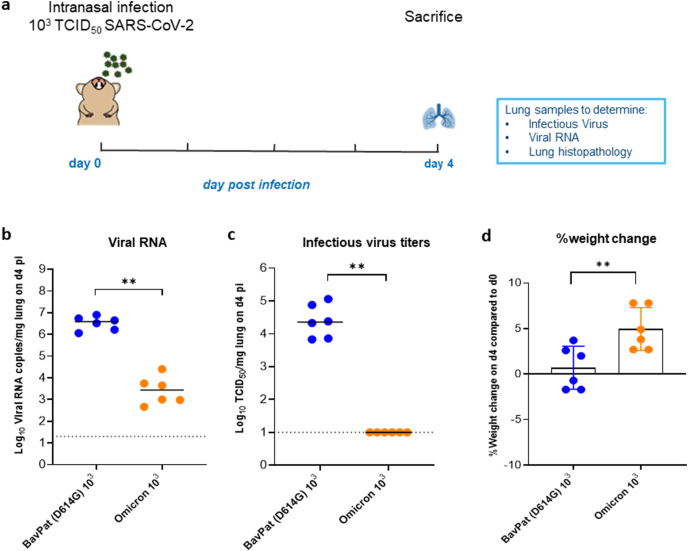
Characterization of the *in vivo* replication of the omicron SARS-CoV-2 variant versus the ancestral D614G strain. (a) Set-up of the Syrian hamster infection study. (b) Viral RNA levels in the lungs of hamsters infected with 10^3^ TCID_50_ of BavPat (D614G) strain (n = 6) or the omicron (B.1.1.529) SARS-CoV-2 variant (n = 6) on day 4 post-infection (pi) are expressed as log_10_ SARS-CoV-2 RNA copies per mg lung tissue. Individual data and median values are presented. (c) Infectious viral loads in the lungs of hamsters infected with the D614G strain or the omicron variant at day 4 pi are expressed as log_10_ TCID_50_ per mg lung tissue. Individual data and median values are presented. (d) Weight change at day 4 pi in percentage, normalized to the body weight at the time of infection. Bars represent means ± SD. Data were analyzed with the Mann−Whitney *U* test, **P < 0.01. All data are from a single experiment.

The ancestral strain used in this study is strain Germany/BavPat1/2020 (also referred to as BavPat-1, EPI_ISL_406,862; 2020-01-28) (Rothe et al., 2020). This strain carries a spike D614G substitution found in early European variants and linked to more efficient transmission (Hou et al., 2021). The omicron (B.1.1.529) variant was isolated from a nasopharyngeal swab taken from a traveler returning to Belgium at the end of November 2021 (hCoV-19/Belgium/rega-20174/2021, EPI_ISL_6,794,907). The virus stocks of the ancestral strain and the omicron variant were grown on Vero E6 cells and the 50% tissue culture infectious doses (TCID_50_) of these stocks were determined by end-point titration on Vero E6 as described before (Boudewijns et al., 2020; Kaptein et al., 2020). The calculated TCID_50_/mL values for the ancestral D614G strain and the omicron variant were 7.07 × 10^5^ and 1.6 × 10^6^, respectively. The hamster infection model of SARS-CoV-2 has been described before (Boudewijns et al., 2020; Kaptein et al., 2020). In Brief, 6–8 weeks old female Syrian hamsters were intranasally infected with 50 μL containing approximately 10^3^ TCID_50_ of either the ancestral strain (BavPat(D614G)) or the omicron VoC (B.1.1.529) SARS-CoV-2 (Fig. 1a). At day four post-infection (4 dpi), animals were euthanized for sampling of the lungs and further analysis by i.p. injection of 500 μL Dolethal (200 mg/mL sodium pentobarbital) (Abdelnabi et al., 2021a). Housing conditions and experimental procedures were approved by the ethics committee of animal experimentation of KU Leuven (license P065-2020).

A median viral RNA load of 4 × 10^6^ RNA copies/mg of lung tissue was detected at 4 dpi in the lungs from the animals infected with the D614G strain (Fig. 1b). On the other hand, ∼3 log_10_ lower viral RNA loads (possibly input only) were detected in the lungs of animals infected with the omicron variant (a median vira RNA load of 3 × 10^3^ RNA copies/mg lung tissue, p = 0.0022, Mann-Whitney Test), Fig. 1b. Infectious virus titers in the lungs of D614G strain-infected animals were around 2 × 10^4^ TCID_50_/mg of lung tissue (Fig. 1c). Strikingly, no infectious virus was detected at all in the lungs of all animals infected with the omicron variant (Fig. 1c, P = 0022 compared to the D614G strain-infected group, Mann-Whitney Test). This is also very different from the other four VoCs that all replicate efficiently and consistently to high viral loads in Syrian hamster lungs (Abdelnabi et al., 2021a, 2021b; Sharma et al., 2021). On the day of sacrifice, animals infected with the omicron variant had gained more body weight (average body weight change from d0 of 3.8%) than the D614G strain-infected animals (average body weight change from d0 of 0.65%), p = 0.0087, Mann-Whitney Test (Fig. 1d).

**Fig. 2:**
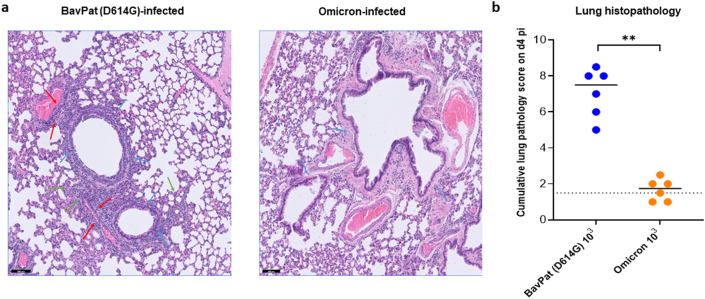
Histopathology of lungs of Syrian hamsters infected with either the D614G strain or the omicron SARS-CoV-2 variant. (a) Representative H&E images of lungs of hamsters infected with 10^3^ TCID_50_ of BavPat (D614G) strain (n = 6) or the omicron (B.1.1.529) SARS-CoV-2 variant at day 4 post-infection (pi). The lungs of hamsters infected with the ancestral D614G strain (left picture) show significant bronchopneumonia (green arrows), perivascular inflammation with peri-vascular oedema (red arrows) and peri-bronchial inflammation (blue arrows), whereas the lungs of the omicron-infected hamsters (Right picture) appear normal with no peri-bronchial inflammation (blue arrows) or bronchopneumonia. Scale bars, 100 μm. (b) Cumulative severity score from H&E stained slides of lungs from hamsters infected with the D614G strain or the omicron variant at day 4 pi. Individual data and median values are presented and the dotted line represents the median score of untreated non-infected hamsters. Data were analyzed with the Mann−Whitney *U* test, **P < 0.01. All data are from a single experiment.

For histological examination, the lungs were fixed overnight in 4% formaldehyde and embedded in paraffin. Tissue sections were analyzed after staining with hematoxylin and eosin and scored blindly for lung damage by an expert pathologist. The scored parameters, to which a cumulative score of 1–3 was attributed, were the following: congestion, intra-alveolar hemorrhagic, apoptotic bodies in bronchus wall, necrotizing bronchiolitis, perivascular oedema, bronchopneumonia, perivascular inflammation, peribronchial inflammation and vasculitis.Hematoxylin/eosin (H&E)-stained images of lungs of hamsters infected with the D614G strain revealed significant pathological signs including peri-bronchial inflammation, bronchopneumonia in the surrounding alveoli and perivascular inflammation with peri-vascular oedema (Fig. 2a). The median cumulative histopathological lung score of the D614G-infected hamsters was 7.5 (Fig. 2b), which is comparable to what we previously reported for this strain (Abdelnabi et al., 2021a). Unlike the D614G strain-infected group, no inflammation or disease signs were observed in the lungs of the omicron-infected animals on day 4 pi (Fig. 2a). The median cumulative histopathological lung scores of the omicron-infected animals was close to the baseline score in untreated, non-infected hamsters (median score of 1.75, Fig. 2b, P = 0022 compared to the D614G group, Mann-Whitney Test).

Taken together, these results clearly demonstrate that the omicron is not able to efficiently replicate in the lower respiratory tract of Syrian hamsters compared to the ancestral D614G strain and other variants of concerns. Similarly, other research groups reported that the viral loads and pathology signs in the lungs of omicron-infected hamsters were much lower compared to the basal strains and the other VoCs (Diamond et al., 2021; McMahan et al., 2022). One possible explanation may be that the heavily mutated spike of the omicron variant is so much better adapted to the human ACE2 receptor hence making the attachment of this variant to the hamster ACE2 less efficient. Interestingly a recent study using human ACE2 transgenic mice (i.e. K18-hACE2 mouse model) revealed that omicron replicates also much less efficiently in nasal tissues and lungs and causes much less severe signs of disease as compared to mice infected with either the ancestral strain or the delta variant (Bentley et al., 2021). In another study, omicron-infected human ACE2 transgenic hamsters also showed reduced mortality and infectious virus loads in the lung compared to those infected with the D614G strain (Diamond et al., 2021). Thus a better adaptation to human ACE2 cannot explain the limited infectivity observed in this humanized mouse model. Another possibility is that the tropism of the omicron is shifted to the upper respiratory tract resulting in limited lung infectivity and hence less also less severe disease. This would be in line with the recently reported data in *ex-vivo* models from which it is evident that the omicron variant is 70-fold more efficient in replicating in human bronchus tissues than the delta variant but much less efficiently replicating in human lung tissues (Chi-wai et al., 2021). The cellular entry of the ancestral strains and that of the non-omicron VoCs depend on binding of the S1 subunit of the Spike (S) protein to the ACE2 receptor (Hoffmann et al., 2020). This process is accompanied by S-protein cleavage (activation) by the transmembrane serine protease 2 (TMPRSS2) to allow the fusion of viral and cellular membranes (Hoffmann et al., 2020). However, recent studies showed that the omicron variant replicates much less efficiently in TMPRSS2-overexpressing cell lines as compared to the delta variant (Meng et al., 2021; Peacock et al., 2022; Zhao et al., 2021). Moreover, unlike the ancestral strains and other VoCs, omicron enters cells more efficiently via the endocytic cathepsin L pathway (Peacock et al., 2022; Zhao et al., 2021). These findings could explain the ability of the omicron variant to replicate better than other variants in the cells of the upper respiratory tract that lack the TMPRSS2 expression. Therefore, further experiments are required to assess the viral loads in lung and other tissues from the upper respiratory tract of omicron-infected hamster at different time points post-infection to explain the limited lung infectivity observed in this study.

The establishment of an efficient preclinical infection model for emerging SARS-CoV-2 variants is pivotal to assess the *in vivo* efficacy of therapeutic and prophylactic interventions (small molecule antivirals and monoclonal antibodies) against these variants. However, because of the limited infectivity of the omicron variant in two well-established SARS-CoV-2 animals models (i.e. in Syrian hamsters and K18-hACE2 mice), this will not be possible for omicron. Given however that drugs and antibodies should ideally be broadly active against any SARS-CoV-2 variant; this should not per se be problematic. Indeed, the efficacy of such therapies will first need to be assessed *in vitro* whereby only therapeutics with equipotent activity should be further considered. In such case, assessment of efficacy in animal infection models could be performed using any of the other VoC as a surrogate for the omicron variant.

## Funding

The authors acknowledge funding by the Flemish Research Foundation (10.13039/501100003130FWO) emergency Covid-19 fund (G0G4820N) and the FWO Excellence of Science (EOS) program (No. 30981113; VirEOS project), the European Union’s Horizon 2020 research and innovation program (No 101003627; SCORE project), the Bill and Melinda Gates Foundation (INV-00636), KU Leuven Internal Funds (C24/17/061), the KU Leuven/UZ Leuven Covid-19 Fund (COVAX-PREC project) and European Health Emergency Preparedness and Response Authority (HERA). X.Z. received funding of the China Scholarship Council (grant No.201906170033). K.D. acknowledges grant support from KU Leuven Internal Funds (C3/19/057 Lab of Excellence).

## Conflict of interest

None to declare.

## Declaration of interests

The authors declare that they have no known competing financial interests or personal relationships that could have appeared to influence the work reported in this paper.

## References

[bib1] Abdelnabi R., Boudewijns R., Foo C.S., Seldeslachts L., Sanchez-Felipe L., Zhang X., Delang L., Maes P., Kaptein S.J.F., Weynand B., Vande Velde G., Neyts J., Dallmeier K. (2021). Comparing infectivity and virulence of emerging SARS-CoV-2 variants in Syrian hamsters. EBioMedicine.

[bib2] Abdelnabi R., Foo C.S., Jochmans D., Vangeel L., De Jonghe S., Augustijns P., Mols R., Weynand B., Wattanakul T., Hoglund R.M., Tarning J., Mowbray C.E., Sjö P., Escudié F., Scandale I., Chatelain E., Neyts J. (2021). The oral protease inhibitor (PF-07321332) protects Syrian hamsters against infection with SARS-CoV-2 variants of concern. bioRxiv.

[bib3] Bentley E.G., Kirby A., Sharma P., Kipar A., Mega D.F., Bramwell C., Penrice-Randal R., Prince T., Brown J.C., Zhou J., Screaton G.R., Barclay W.S., Owen A., Hiscox J.A., Stewart J.P. (2021). SARS-CoV-2 Omicron-B.1.1.529 Variant leads to less severe disease than Pango B and Delta variants strains in a mouse model of severe COVID-19. bioRxiv.

[bib4] Boudewijns R., Thibaut H.J., Kaptein S.J.F., Li R., Vergote V., Seldeslachts L., Van Weyenbergh J., De Keyzer C., Bervoets L., Sharma S., Liesenborghs L., Ma J., Jansen S., Van Looveren D., Vercruysse T., Wang X., Jochmans D., Martens E., Roose K., De Vlieger D., Schepens B., Van Buyten T., Jacobs S., Liu Y., Martí-Carreras J., Vanmechelen B., Wawina-Bokalanga T., Delang L., Rocha-Pereira J., Coelmont L., Chiu W., Leyssen P., Heylen E., Schols D., Wang L., Close L., Matthijnssens J., Van Ranst M., Compernolle V., Schramm G., Van Laere K., Saelens X., Callewaert N., Opdenakker G., Maes P., Weynand B., Cawthorne C., Vande Velde G., Wang Z., Neyts J., Dallmeier K. (2020). STAT2 signaling restricts viral dissemination but drives severe pneumonia in SARS-CoV-2 infected hamsters. Nat. Commun..

[bib5] Chi-wai M. (2021).

[bib6] del Rio C., Omer S.B., Malani P.N. (2021). Winter of Omicron—The Evolving COVID-19 Pandemic. JAMA.

[bib7] Diamond M., Halfmann P., Maemura T., Iwatsuki-Horimoto K., Iida S., Kiso M., Scheaffer S., Darling T., Joshi A., Loeber S., Foster S., Ying B., Whitener B., Floyd K., Ujie M., Nakajima N., Ito M., Wright R., Uraki R., Li R., Sakai Y., Liu Y., Larson D., Osorio J., Hernandez-Ortiz J., ÄŒiuoderis K., Florek K., Patel M., Bateman A., Odle A., Wong L.-Y., Wang Z., Edara V.V., Chong Z., Thackray L., Ueki H., Yamayoshi S., Imai M., Perlman S., Webby R., Seder R., Suthar M., Garcia-Sastre A., Schotsaert M., Suzuki T., Boon A., Kawaoka Y., Douek D., Moliva J., Sullivan N., Gagne M., Ransier A., Case J., Jeevan T., Franks J., Fabrizio T., DeBeauchamp J., Kercher L., Seiler P., Singh G., Warang P., Gonzalez-Reiche A.S., Sordillo E., van Bakel H., Simon V. (2021). The SARS-CoV-2 B.1.1.529 Omicron virus causes attenuated infection and disease in mice and hamsters. Res. Sq.

[bib8] Hoffmann M., Kleine-Weber H., Schroeder S., Krüger N., Herrler T., Erichsen S., Schiergens T.S., Herrler G., Wu N.H., Nitsche A., Müller M.A., Drosten C., Pöhlmann S. (2020). SARS-CoV-2 cell entry depends on ACE2 and TMPRSS2 and is blocked by a clinically proven protease inhibitor. Cell.

[bib9] Hou Y.J., Chiba S., Halfmann P., Ehre C., Kuroda M., Dinnon K.H., Leist S.R., Schäfer A., Nakajima N., Takahashi K., Lee R.E., Mascenik T.M., Graham R., Edwards C.E., Tse L.V., Okuda K., Markmann A.J., Bartelt L., Silva A., De, Margolis D.M., Boucher R.C., Randell S.H., Suzuki T., Gralinski L.E., Kawaoka Y., Baric R.S. (2021). SARS-CoV-2 D614G variant exhibits efficient replication ex vivo and transmission in vivo. Science.

[bib10] Kaptein S.J.F., Jacobs S., Langendries L., Seldeslachts L., ter Horst, S., Liesenborghs, L., Hens, B., Vergote, V., Heylen, E., Barthelemy, K., Maas, E., de Keyzer, C., Bervoets, L., Rymenants, J., van Buyten, T., Zhang, X., Abdelnabi, R., Pang, J., Williams, R., Thibaut, H.J., Dallmeier, K., Boudewijns, R., Wouters, J., Augustijns, P., Verougstraete, N., Cawthorne, C., Breuer, J., Solas, C., Weynand, B., Annaert, P., Spriet, I., Velde, G. Vande, Neyts, J., Rocha-Pereira, J., Delang, L (2020). Favipiravir at high doses has potent antiviral activity in SARS-CoV-2−infected hamsters, whereas hydroxychloroquine lacks activity. Proc. Natl. Acad. Sci. U. S. A.

[bib11] Karim S.S.A., Karim Q.A. (2021). Omicron SARS-CoV-2 variant: a new chapter in the COVID-19 pandemic. Lancet.

[bib12] McMahan K., Giffin V., Tostanoski L.H., Chung B., Siamatu M., Suthar M.S., Halfmann P., Kawaoka Y., Piedra-Mora C., Martinot A.J., Kar S., Andersen H., Lewis M.G., Barouch D.H. (2022). Reduced pathogenicity of the SARS-CoV-2 omicron variant in hamsters. bioRxiv.

[bib13] Meng B., Ferreira I.A.T.M., Abdullahi A., Saito A., Kimura I., Yamasoba D., Kemp S.A., Goonawardane N., Papa G., Fatihi S., Rathore S., Ikeda T., Toyoda M., Tan T.S., Kuramochi J., Mitsunaga S., Ueno T., Charles O.J. (2021). SARS-CoV-2 Omicron spike mediated immune escape, infectivity and cell-cell fusion. bioRxiv.

[bib14] Peacock T.P., Brown J.C., Zhou J., Thakur N., Newman J., Kugathasan R., Sukhova K., Kaforou M., Bailey D., Barclay W.S. (2022). The SARS-CoV-2 variant, Omicron, shows rapid replication in human primary nasal epithelial cultures and efficiently uses the endosomal route of entry. bioRxiv.

[bib15] Plante J.A., Mitchell B.M., Plante K.S., Debbink K., Weaver S.C., Menachery V.D. (2021). The variant gambit: COVID’s next move. Cell Host Microbe.

[bib16] Rothe C., Schunk M., Sothmann P., Bretzel G., Froeschl G., Wallrauch C., Zimmer T., Thiel V., Janke C., Guggemos W., Seilmaier M., Drosten C., Vollmar P., Zwirglmaier K., Zange S., Wölfel R., Hoelscher M. (2020). Transmission of 2019-nCoV infection from an asymptomatic contact in Germany. N. Engl. J. Med..

[bib17] Sharma S., Vercruysse T., Sanchez-Felipe L., Kerstens W., Abdelnabi R., Foo C., Lemmens V., Van Looveren D., Maes P., Baele G., Weynand B., Lemey P., Neyts J., Thibaut H.J., Dallmeier K. (2021). Universal COVID-19 vaccine with updated spike antigen confers full protection against all SARS-CoV-2 variants of concern. bioRxiv.

[bib18] World Health Organization (2021). Classification of omicron (B.1.1.529): SARS-CoV-2 variant of concern. J. Med. Virol..

[bib19] Zhao H., Lu L., Peng Z., Chen L.-L., Meng X., Zhang C., Ip J.D., Chan W.-M., Chu A.W.-H., Chan K.-H., Jin D.-Y., Chen H., Yuen K.-Y., W K.K. (2021). SARS-CoV-2 Omicron variant shows less efficient replication and fusion activity when compared with delta variant in TMPRSS2-expressed cells. Emerg. Microb. Infect..

